# Quantification of Sunscreen Benzophenone-4 in Hair Shampoos by Hydrophilic Interactions Thin-Layer Chromatography/Densitometry or Derivative UV Spectrophotometry

**DOI:** 10.1155/2015/695658

**Published:** 2015-02-04

**Authors:** Anna W. Sobańska, Katarzyna Kałębasiak, Jarosław Pyzowski, Elżbieta Brzezińska

**Affiliations:** Department of Analytical Chemistry, Medical University of Lodz, Ulica Muszynskiego 1, 90-151 Lodz, Poland

## Abstract

Benzophenone-4 (BZ4) was separated from surfactants, dyes, preservatives, and other components of hair shampoos by thin-layer chromatography on silica gel 60 stationary phase, with ethyl acetate-ethanol-water-pH 6 phosphate buffer (15 : 7 : 5 : 1 v/v/v/v) as mobile phase. Densitometry scanning of chromatograms was performed at 285 nm. The densitometric calibration curve for BZ4 was nonlinear (second-degree polynomial), with *R* > 0.999. The limits of detection and quantification were *ca.* 0.03 and *ca.* 0.1 *μ*g spot^−1^, respectively. The results obtained by HPTLC-densitometry were compared to those obtained by zero and 2nd derivative UV spectrophotometry. In the case of spectrophotometric methods, calibration curves were linear with *R* > 0.9998. The chromatographic method was fully validated.

## 1. Introduction

**Figure 1:**
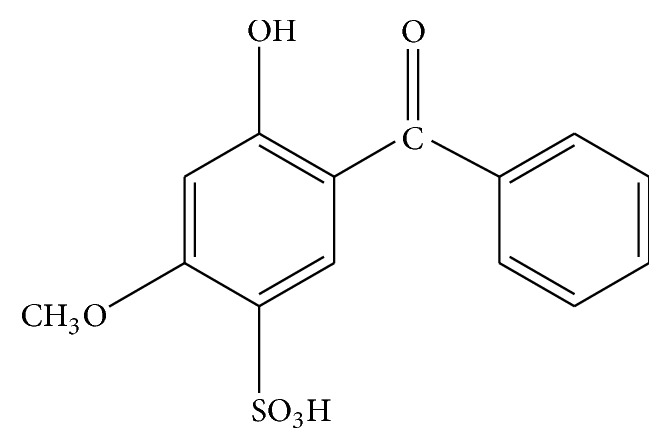
Structure of BZ4.

Benzophenone (BZ4, Figure 1) is a water-soluble UVB filter (*λ*_max⁡_ in methanol 285 nm) manufactured by BASF under the trademark Uvinul MS40 and used in cosmetic formulations at concentrations up to 5% (as free acid). Because of its highly acidic character, it must be neutralized with sodium hydroxide or triethanolamine prior to addition to a cosmetic formulation. Due to its good water and alcohol solubility, it is commonly used in aqueous preparations (shampoos, oil-free sunscreen sprays) or to protect cosmetic products including perfumes against photodegradation [1].

Benzophenone-4 was quantified in cosmetic products mainly by RP-HPLC [2–9] or, less frequently, SI (sequential injection) analysis [10] or CE (capillary electrophoresis) [11, 12]. Liquid chromatography was also used in BZ4 quantification in environmental samples [13, 14] or in skin permeation studies [15]. In such studies, a radioanalytical method involving BZ4 labeled with radioisotope ^14^C (at carbonyl group) was also applied [16].

The objective of this study was to propose a cheap and convenient method of quantification of benzophenone-4 in aqueous cosmetic preparations by hydrophilic interaction thin-layer chromatography followed by densitometry and, alternatively, by UV spectrophotometry.

## 2. Experimental

### 2.1. Chemicals, Material, and Solutions

Uvinul MS40 (benzophenone-4) was kindly donated by BASF. Ethyl acetate, ethanol, methanol, potassium phosphate monobasic, and sodium phosphate dibasic were from Polskie Odczynniki Chemiczne (POCh), Gliwice, Poland. Hair shampoos containing benzophenone-4 and benzophenone-free (blank shampoo) were purchased locally. All shampoos analyzed throughout this study were preserved with sodium benzoate, bronopol, or thiazolinone derivatives and contained sodium laureth sulfate, cocamide DEA, dyes (CI 19140, CI 42080, or CI 17200), vitamins, fragrances, and plant extracts (chamomile, lavender, cornflower, and wild rose). All chemicals used in this study were of analytical quality. pH 6.0 phosphate buffer was prepared according to [17].

#### 2.1.1. Method I

Uvinul MS40, 500 mg, was weighed accurately into a 100 mL volumetric flask, dissolved in 16.2 mL of 0.1 mol L^−1^ aqueous NaOH, and diluted to volume with water to give a stock solution I of the concentration 5 mg mL^−1^. The appropriate volumes of BZ4 stock solution were pipetted to 25 mL volumetric flasks, pH 6 phosphate buffer was added (2 mL), and water was added to volume to furnish standard solutions of BZ4 (0.1, 0.2, 0.4, 0.6, 0.8, 1.0, 1.2, 1.4, 1.6, 1.8, and 2.0 mg mL^−1^, BZ4 expressed as free acid).

#### 2.1.2. Method II

2 mL of BZ4 stock solution I was diluted to 100 mL with water to furnish a stock solution II (0.1 mg mL^−1^). The appropriate volumes of stock solution II were transferred to 25 mL volumetric flasks, pH 6 phosphate buffer (5 mL) was added, and the solutions were diluted to volume with water to furnish solutions of BZ4 of concentrations 0.004, 0.008, 0.016, 0.024, 0.032, and 0.040 mg mL^−1^ (BZ4 expressed as free acid).

### 2.2. Sample Preparation

#### 2.2.1. Method I

Shampoo in the quantity corresponding to* ca.* 1–5 mg BZ4 was weighed accurately into 10 mL volumetric flasks and diluted with pH 6 phosphate buffer (1 mL) and water to volume.

#### 2.2.2. Method II

Shampoo in the quantity corresponding to* ca*. 0.5–2.5 mg BZ4 was weighed accurately into 100 mL flasks and diluted with pH 6 phosphate buffer (5 mL) and water to volume.

### 2.3. Thin-Layer Chromatography/Densitometry

Thin-layer chromatography was performed on 10 × 10 cm HP quality silica gel 60 plates (layer thickness 0.2 mm) from Merck or on 10 × 20 cm standard quality silica gel 60 plates (layer thickness 0.25 mm), also from Merck. Plates were developed with methanol-dichloromethane 1 : 1 (v/v) and dried at room temperature overnight prior to use. Standard solutions prepared for Method I (Section 2.1) were spotted with the Desaga AS30 sampler equipped with a 10 *μ*L syringe (1 *μ*L spot^−1^), 15 mm from the from the plate bottom edge, and at 8 mm intervals, starting 10 mm from the plate edge and developed with ethyl acetate-ethanol-water-pH 6 phosphate buffer 15 : 7 : 5 : 1 (v/v/v/v) as mobile phase. Plates were developed in a vertical chromatographic chamber lined with filter paper and previously saturated with the mobile phase vapor for 20 min. Development distance was 75 mm from the plate bottom edge. After development, plates were dried at room temperature (20°C), scanned, and analyzed in reflectance mode with the Desaga CD 60 densitometer at 285 nm (*R*_*f*_ = 0.78).

### 2.4. UV Spectrophotometry

Spectroscopic measurements were performed with Lambda 25 UV/VIS spectrophotometer, Perkin-Elmer. Standard solutions of BZ4 prepared according to Section 2.1. (Method II) were placed in 1 cm quartz glass cuvettes and scanned over the wavelength range 200–420 nm.

### 2.5. Chromatographic Quantification of Benzophenone-4 in Shampoos

The shampoo solutions in buffered water (Method I) prepared as described above (Section 2.2) were spotted on silica gel 60 HPTLC plates (1–10 *μ*L spot^−1^ depending on the expected BZ4 concentration). The plates were then chromatographed as described above for BZ4 standards (Section 2.3).

### 2.6. Spectrophotometric Quantification of Benzophenone-4 in Shampoos

The shampoo solutions in buffered water (Method II) prepared as described above (Section 2.2) were placed in 1 cm quartz glass cuvettes and scanned with the Lambda 25 UV/VIS spectrophotometer as described above (Section 2.4.).

## 3. Results and Discussion

### 3.1. Method Development

#### 3.1.1. Chromatography and Densitometry

**Figure 2:**
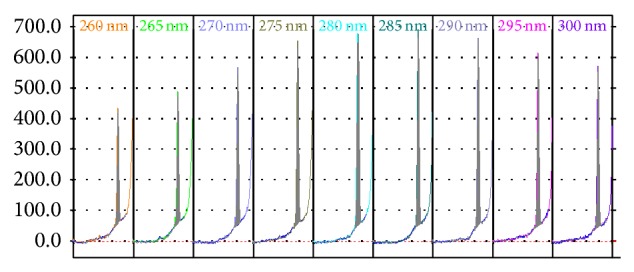
Multiwavelength scans for BZ4, silica gel 60, ethyl acetate-ethanol-water-pH 6 phosphate buffer 14 : 7 : 5 : 1 (v/v/v/v), scanning in reflectance mode.

The sun-care preparations analyzed in this study contained benzophenone-4 and surfactants, plant extracts, preservatives, and other ingredients that may potentially absorb light within the same range as BZ4. On the basis of our earlier research [19], it was decided that silica gel 60 is the stationary phase of choice and the most effective mobile phase capable of BZ4 separation from other cosmetic ingredients was ethyl acetate-ethanol-water-pH 6 phosphate buffer 14 : 7 : 5 : 1 (v/v/v/v). Analytical wavelength suitable for BZ4 analysis (285 nm) was selected on the basis of multiwavelength scans obtained for this sunscreen (Figure 2).

#### 3.1.2. UV Spectrophotometry

**Figure 3:**
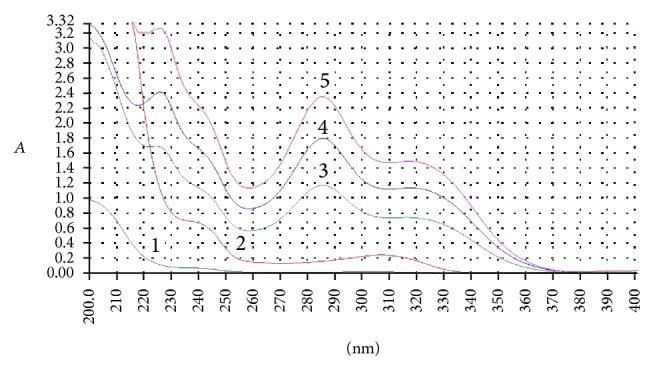
Selected UV spectra of shampoo solutions and BZ4 standards in pH 6 phosphate buffer-water 1 : 20 (v/v). 1: shampoo matrix (2.5 mg mL^−1^, 0% BZ4); 2: shampoo matrix (20 mg mL^−1^, 0% BZ4); 3: shampoo (2.5 mg mL^−1^, 1.0% BZ4); 4: BZ4 standard (0.04 mg mL^−1^); 5: shampoo (2.5 mg mL^−1^, 2.0% BZ4).

**Figure 4:**
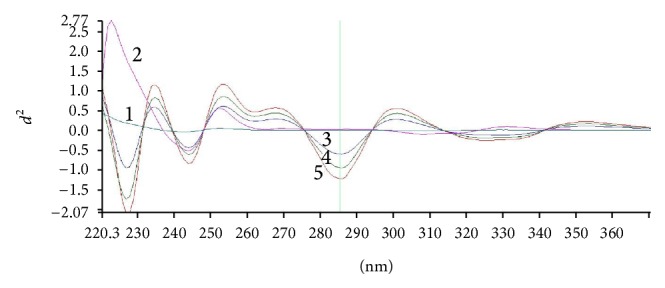
Selected 2nd derivative UV spectra of shampoo solutions and BZ4 standards in pH 6 phosphate buffer-water 1 : 20 (v/v). 1: shampoo matrix (2.5 mg mL^−1^, 0% BZ4); 2: shampoo matrix (20 mg mL^−1^, 0% BZ4); 3: shampoo (2.5 mg mL^−1^, 1.0% BZ4); 4: BZ4 standard (0.04 mg mL^−1^); 5: shampoo (2.5 mg mL^−1^, 2.0% BZ4).

The cosmetic preparations under investigation contained raw materials other than BZ4 (such as cosmetic dyes) that may absorb UV light within the same range as BZ4, thus influencing the accuracy of the analysis (see Figure 3, curves 1 and 2, solutions of a typical shampoo matrix containing 0% BZ4). One of the most convenient methods used to reduce matrix effects in UV/VIS spectrophotometry is derivative spectroscopy with the 2nd derivative being the most effective tool capable of matrix effects suppression. After the analysis of 2nd derivative spectra for BZ4 standards and the shampoo matrix (Figure 4), it was decided that the analytical wavelength of choice is in this case 285 nm (at this wavelength, the 2nd derivative of absorbance for the shampoo matrix is very close to 0 even at higher concentrations and for BZ4 samples and standards there is a clear maximum of the *d* ^2^ *A*/*dλ* ^2^ curve).

### 3.2. Chromatographic Method Validation

#### 3.2.1. Specificity

**Figure 5:**
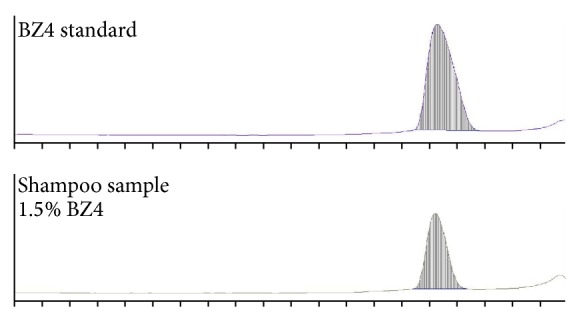
Densitogram of BZ4 standard (1.8 *μ*g) and shampoo sample (1.5% BZ4).

**Figure 6:**
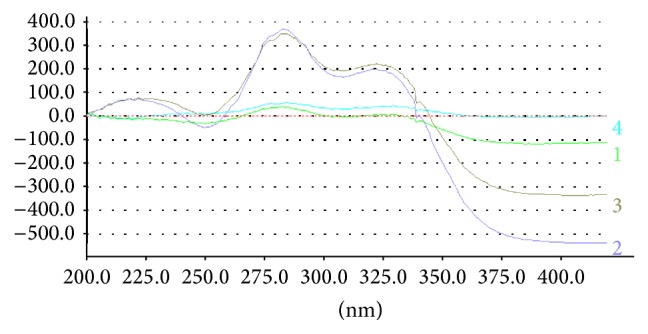
UV spectra of BZ4 standard (1, 2) and shampoo sample (3, 4) collected directly from the chromatographic plate in the reflectance mode.

Identity of chromatographic spots for BZ4 isolated from the shampoo by thin-layer chromatography was tested by comparison of their densitograms with those obtained for the BZ4 standard solutions (Figure 5). Further investigations of the identity and purity of spots were based on the analysis of the UV spectra of BZ4 isolated from the shampoo and the BZ4 standards. Spectra were collected directly from the chromatographic plates in the reflectance mode (for each spot three spectra were collected, for the spot center and both edges; in Figure 6 only two spectra are shown for each spot for the sake of clarity).

#### 3.2.2. Calibration

**Figure 7:**
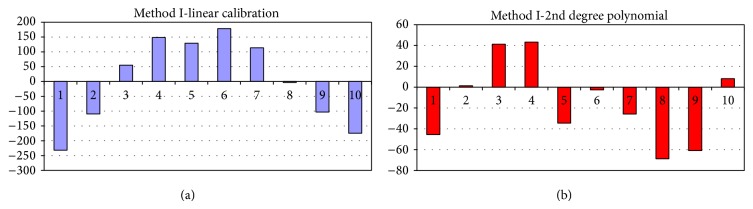
Analysis of residues for the linear (a) and no-linear (b) calibration plot, chromatographic method. Plot (a): a clearly visible trend. Plot (b): no particular trend.

The densitometric calibration plot was obtained by plotting peak areas against the amount of BZ4 over the range 0.1–2.0 *μ*g spot^−1^. The nonlinearity of the calibrating plot was visible. The densitometric calibration plot was finally generated in the form of the second-degree polynomial (Table 1) and its quality was assessed by means of *R* values and nonnumerical analysis of residues according to [20] (Figure 7).

**Figure 8:**
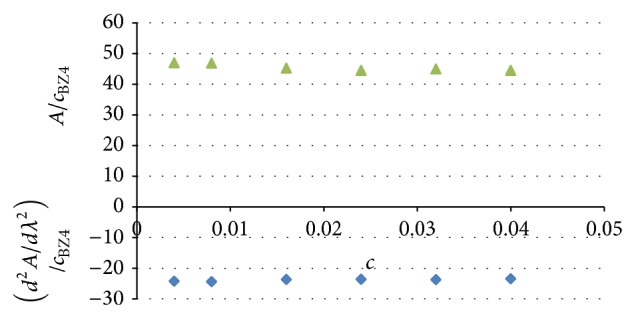
Linearity analysis for spectrophotometric calibration plots: analytical signal-to-analyte concentration versus concentration, according to [18]. All absorbances were measured in triplicate.

The spectrophotometric calibration plots were linear within the studied concentration range (Table 1). Their linearity was checked according to [18] (Figure 8). The general method for linearity testing proposed in this reference is based on the analysis of the ratio of the analytical signal (**y**) to the analyte concentration (**x**). If the signal-concentration relationship is linear, **y**/**x** is constant (within an acceptable range, usually ±5%). When this approach was applied to the linear calibration plots generated during this study, all values of signal-to-concentration ratio were within acceptable limits for particular plots (45.54 ± 3.3% absorbance and −23.85 ± 2.2% for the 2nd derivative of absorbance, resp.).

#### 3.2.3. Precision

Repeatability of chromatographic method was tested according to [19, 20] by replicating the entire method (Section 2.5) on the same day, using the same cosmetic preparations, batches of solvents, and chromatographic plates, by the same analyst (Day 1, Analysis A and Analysis B). Intermediate precision was verified according to [19, 20] by repeating the procedure on the same cosmetic preparations but on a different day, by a different analyst, using other batches of solvents and chromatographic plates (Day 2). The results of these experiments (Table 2) prove that the methods precision is sufficient for routine product analysis.

#### 3.2.4. Limits of Detection and Quantification

The instrumental detection limits (IDL) and instrumental quantification limits (IQL) for chromatographic method were determined experimentally on the basis of signal-to-noise (*S*/*N*) ratio according to [18]. Blank solutions of pH 6 phosphate buffer containing zero concentration of BZ4 were analyzed in triplicate along with the series of BZ4 standards according to the procedure described in Section 2.3. The blank signals obtained in this manner were used as the baseline noise. The IDL and IQL were calculated as the concentrations that yielded peaks with *S*/*N* of 3 and 10, respectively. The results of these determinations expressed as the amount of BZ4 per spot are given in Table 1. In order to estimate the method LOQ, the minimum concentration of BZ4 that can be realistically quantified in the actual cosmetic preparations, the blank shampoo samples were fortified with BZ4 at the following concentrations: 0.01, 0.02, 0.04, and 0.06% (w/w), diluted with pH 6 phosphate buffer and analyzed in triplicate as described in Section 2.5. In order to avoid excessive viscosity and foaming of the sample solutions, their maximum concentration was 100 mg mL^−1^ and due to the capacity of the applicator syringe, the maximum application volume was 10 *μ*L. As the result of these experiments, it was estimated that the lower quantification limit for BZ4 in shampoos by thin-layer chromatography/densitometry without samples preconcentration is* ca*. 0.02% (w/w).

Similarly, limits of detection/quantification were estimated for the spectroscopic methods. Instrumental limits of detection/quantification (IDL and IQL) were calculated from the signal-to-noise ratio (Table 1) and the limit of quantification (LOQ) for the entire 2nd derivative spectroscopic method was studied on the basis of the same shampoo samples containing 0.01, 0.02, 0.04, and 0.06% BZ4 (w/w). Samples were diluted with pH 6 phosphate buffer and water (10 mg mL^−1^, higher concentrations should be avoided because of increasing matrix effects) and the estimated LOQ in shampoos was* ca*. 0.05% (w/w).

#### 3.2.5. Robustness

After due consideration of factors that can influence the analysis results, it was concluded that the critical points are the quality of chromatographic plates (HPTLC versus TLC), the method of spotting, and the pH of the mobile phase. The same cosmetic preparations were analyzed on HPTLC silica gel 60 chromatographic plates with automatic spotting and on standard TLC silica gel 60 plates with manual spotting with a Hamilton microsyringe. The mobile phase ethyl acetate-ethanol-water-pH 6 phosphate buffer 15 : 7 : 5 : 1 (v/v/v/v) was replaced with ethyl acetate-ethanol-water 15 : 7 : 6 (v/v/v). The results of these analyses (Table 2) are similar but coefficients of variations are, as it may have been expected, slightly higher for manual spotting.

#### 3.2.6. Accuracy

Blank shampoo was spiked with BZ4 at five concentrations: 0.5, 1.0, 1.5, 2.0, and 5.0% (w/w) corresponding to 0.25, 0.50, 0.75, and 1.0 *μ*g spot^−1^ (1 *μ*L spot^−1^ of the shampoo solution prepared according to Section 2.2, 50 mg of shampoo per 1 mL of sample solution) and 1.25 *μ*g spot^−1^ (1 *μ*L spot^−1^ of the shampoo solution prepared according to Section 2.2, 25 mg of shampoo per 1 mL of sample solution), respectively. The chromatographic procedure described in Section 2 was performed on the samples and the recoveries are presented in Table 3. Spectrophotometric determination of BZ4 in the same spiked shampoos was performed according to Section 2.6 (sample solutions were prepared according to Section 2.3, 2.5 or 1.0 mg of shampoo per 1 mL of sample solution) and the results are given in Table 3.

#### 3.2.7. Storage and Stability of Standard Solutions

Standard solutions of BZ4 used in this investigation were refrigerated between the experiments and not exposed to light except for time needed for plates spotting. The stability of all solutions was in these conditions excellent over at least two weeks.

## 4. Conclusions

Benzophenone-4 may be effectively separated from other cosmetic ingredients (dyes, preservatives, vitamins, and plant actives) by hydrophilic interaction high performance thin-layer chromatography on silica gel 60 with ethyl acetate-ethanol-water-pH 6 phosphate buffer as mobile phase. Densitometric quantification of BZ4 at 285 nm is fast, reliable, and cost-effective and it may be recommended for routine analysis of shampoos containing BZ4 at different levels of concentrations (concentrations of the shampoo solutions and volumes per spot may be adjusted to the needs over a relatively broad range of BZ4 concentration in a cosmetic formulation). The results of chromatographic/densitometric determination (precision, accuracy) are superior to those of spectrophotometric analysis by zero derivative spectroscopy and similar to those obtained by 2nd derivative spectroscopic method but the flexibility of the chromatographic method is higher; the matrix effects do not pose a problem and the limit of quantification is slightly lower than that of the spectrophotometric analysis.

## Figures and Tables

**Table 1:** Parameters of calibration plots.

	Chromatography/densitometry	Spectrophotometric 0 derivative 285 nm	Spectrophotometric 2nd derivative 285 nm
Calibration plot	*y* = −424.79*x* ^2^ + 2639.7*x* + 213.18	*y* = 44.44*x* + 0.0083	*y* = −23.403*x* − 0.0055
*R* ^2^	0.9990	0.9999	0.9999
IDL	0.03 *μ*g spot^−1^	0.001 mg mL^−1^	0.001 mg mL^−1^
IQL	0.1 *μ*g spot^−1^	0.003 mg mL^−1^	0.003 mg mL^−1^

**Table 2:** Chromatographic determinations of BZ4 in the commercial shampoo (10 *µ*L spot^−1^, sample concentration 10 mg mL^−1^), *n* = 3. Conditions: Section 2.5 (“Day 1,” “Day 2”); Section 3.2.5. (“Changed parameters”).

	Day 1		Day 2	Changed parameters
	Analysis A	Analysis B		
Received	0.60 *µ*g	0.62 *µ*g	0.61 *µ*g	0.62 *µ*g
% BZ4 in shampoo	0.06%	0.06%	0.06%	0.06%
CV%	1.26	3.04	1.33	6.26

**Table 3:** Recovery studies.

% BZ4 in spiked shampoo	Chromatographic		Spectrophotometric		
				0 derivative 285 nm	2nd derivative 285 nm
0.5	Theoretical *µ*g spot^−1^	0.25^a^	Shampoo sample concentration mg mL^−1^	2.5	2.5
	Experimental *µ*g spot^−1^	0.25	Experimental % BZ4 in shampoo	0.53	0.51
	Recovery %	100.0	Recovery %	106.7	102.0
	CV% (*n* = 3)	1.99	CV% (*n* = 3)	0.62	0.70

1.0	Theoretical *µ*g spot^−1^	0.50^a^	Shampoo sample concentration mg mL^−1^	2.5	2.5
	Experimental *µ*g spot^−1^	0.49	Experimental % BZ4 in shampoo	1.05	1.02
	Recovery %	98.0	Recovery %	104.7	102.0
	CV% (*n* = 3)	1.10	CV% (*n* = 3)	0.87	0.81

1.5	Theoretical *µ*g spot^−1^	0.75^a^	Shampoo sample concentration mg mL^−1^	2.5	2.5
	Experimental *µ*g spot^−1^	0.77	Experimental % BZ4 in shampoo	1.59	1.53
	Recovery %	102.7	Recovery %	106.1	102.6
	CV% (*n* = 3)	1.61	CV% (*n* = 3)	0.88	0.67

2.0	Theoretical *µ*g spot^−1^	1.00^a^	Shampoo sample concentration mg mL^−1^	2.5	2.5
	Experimental *µ*g spot^−1^	1.03	Experimental % BZ4 in shampoo	2.10	2.06
	Recovery %	103.0	Recovery %	105.1	103.2
	CV% (*n* = 3)	0.58	CV% (*n* = 3)	1.03	1.05

5.0	Theoretical *µ*g spot^−1^	1.25^b^	Shampoo sample concentration mg mL^−1^	1.0	1.0
	Experimental *µ*g spot^−1^	1.27	Experimental % BZ4 in shampoo	5.32	5.07
	Recovery %	102.0	Recovery %	106.4	101.4
	CV% (*n* = 3)	1.33	CV% (*n* = 3)	1.54	1.41

^a^Shampoo sample concentration—50 mg mL^−1^.

^b^Shampoo sample concentration—25 mg mL^−1^.
